# An efficient and high-throughput method for the evaluation of mitochondrial dysfunction in frozen brain samples after traumatic brain injury

**DOI:** 10.3389/fmolb.2024.1378536

**Published:** 2024-06-05

**Authors:** Hemendra J. Vekaria, Olivia J. Kalimon, Paresh Prajapati, Gopal V. Velmurugan, Patrick G. Sullivan

**Affiliations:** ^1^ Spinal Cord and Brain Injury Research Center, College of Medicine, University of Kentucky, Lexington, KY, United States; ^2^ Lexington VA Medical Center, United States Department of Veterans Affairs, Lexington, KY, United States; ^3^ Department of Neuroscience, College of Medicine, University of Kentucky, Lexington, KY, United States; ^4^ Sanders-Brown Center on Aging, University of Kentucky College of Medicine, Lexington, KY, United States; ^5^ Department of Physiology, College of Medicine, University of Kentucky, Lexington, KY, United States

**Keywords:** electron transport chain, respiration, mitochondria, traumatic brain injury, OxPhos, Seahorse assay, frozen tissue

## Abstract

Mitochondrial function analysis is a well-established method used in preclinical and clinical investigations to assess pathophysiological changes in various disease states, including traumatic brain injury (TBI). Although there are multiple approaches to assess mitochondrial function, one common method involves respirometric assays utilizing either Clark-type oxygen electrodes or fluorescent-based Seahorse analysis (Agilent). However, these functional analysis methods are typically limited to the availability of freshly isolated tissue samples due to the compromise of the electron transport chain (ETC) upon storage, caused by freeze–thaw-mediated breakdown of mitochondrial membranes. In this study, we propose and refine a method for evaluating electron flux through the ETC, encompassing complexes I, II, and IV, in frozen homogenates or mitochondrial samples within a single well of a Seahorse plate. Initially, we demonstrate the impact of TBI on freshly isolated mitochondria using the conventional oxidative phosphorylation protocol (OxPP), followed by a comparison with ETC analysis conducted on frozen tissue samples within the context of a controlled cortical impact (CCI) model of TBI. Additionally, we explore the effects of mitochondrial isolation from fresh *versus* snap-frozen brain tissues and their storage at −80°C, assessing its impact on electron transport chain protocol (ETCP) activity. Our findings indicate that while both sets of samples were frozen at a single time point, mitochondria from snap-frozen tissues exhibited reduced injury effects compared to preparations from fresh tissues, which were either homogenized or isolated into mitochondria and subsequently frozen for later use. Thus, we demonstrate that the preparation of homogenates or isolated mitochondria can serve as an appropriate method for storing brain samples, allowing for later analysis of mitochondrial function, following TBI using ETCP.

## Introduction

Mitochondrial dysfunction is a pivotal mechanism contributing to metabolic dysfunction, following experimental traumatic brain injury (TBI) and other neurological disorders, including Alzheimer’s and Parkinson’s diseases (Lyons et al., 2018; Grewal et al., 2020; Crivelli et al., 2023). Beyond their primary role in ATP generation, mitochondria serve as pivotal regulators involved in diverse cellular processes. Their involvement in calcium buffering, lipid synthesis, beta-oxidation, immune responses, and apoptosis underscores their significance in regulating cellular metabolism, disease progression, and health (Duchen, 2000; Sullivan et al., 2002; Pandya et al., 2013; Pandya et al., 2015; Houten et al., 2016). Given the instrumental role mitochondria play in these key metabolic pathways, the significance of mitochondrial function becomes prominent in comprehending pathological changes, with a particular emphasis on TBI (Sullivan et al., 2002; Sullivan et al., 2005; Singh et al., 2006; Hubbard et al., 2019b; Vekaria et al., 2020; Pandya et al., 2023). Evaluating mitochondrial function has become integral in the preclinical assessment of many pathological conditions and there is a growing emphasis on early testing of mitochondrial impact in the drug development process to mitigate failure rates during both preclinical and clinical phases (Gohil et al., 2010; Yonutas et al., 2016; Lamade et al., 2019; Finlin et al., 2021; Hubbard et al., 2021).

Among all mitochondrial functions, the ETC stands out as a key component crucial for generating membrane potential and subsequent ATP production, which undergoes subtle changes in diverse pathological conditions. The gold standard for the comprehensive assessment of mitochondrial function has relied on respirometric flux analysis, employing an oxygen Clark-type electrode, allowing the determination of energy homeostasis under selective conditions (Pandya et al., 2009; Vekaria et al., 2017; Hubbard et al., 2019a). A significant constraint in employing flux analysis lies in the strict necessity for functional mitochondria, typically requiring mitochondria to be freshly isolated from live biological samples (Nukala et al., 2006; Hubbard et al., 2019a). To overcome the use of only freshly isolated mitochondria, alternative approaches for using the frozen stored samples involve the assessment of individual metabolic mitochondrial ETC components using various reductionist approaches, such as employing biochemical reaction kinetics measurements with selective enzyme assays (Pandya et al., 2023) that employ fluorescence- or absorbance-based plate reader assays (Pandya et al., 2019), observing protein or gene expression changes using molecular biology tools such as Western blot or qPCR (Acin-Perez et al., 2004), or studying enzyme supra-complexes through techniques like blue native polyacrylamide gel electrophoresis (BN-PAGE). However, these methods pose significant limitations, including time consumption, substantial sample requirements, and the expense of procuring separate reagents/kits for each assay. All these methods rely on the measurement of non-physiological electron donors, which may influence the actual activities of the ETC function. Moreover, variability in assay conditions, reading parameters, and multiple procedural steps introduced inconsistencies in determining ETC activity.

In recent times, substantial strides have been made to enhance the throughput and efficiency of mitochondrial functional analysis using the Seahorse (Agilent) technology. Although these assays have been predominantly conducted with live cells or freshly isolated healthy mitochondria to examine ETC-mediated cellular O_2_ consumption rates, the application of this technology to frozen tissue samples remains limited. Cryopreserved samples, unfortunately, pose a challenge due to the compromised membrane integrity of mitochondria, resulting in decreased standard respiratory rates upon thawing (Kuznetsov et al., 2003; Nukala et al., 2006; García-Roche et al., 2018). Recently, it has been shown that frozen mitochondria can still be investigated by supplementing the leaked cytochrome C to complete the flow of ETC to measure oxygen as a final electron acceptor, as in intact mitochondria (Acin-Perez et al., 2020; Jaber et al., 2020; Acin-Perez et al., 2021).

In the current study, we have designed and validated a straightforward, expeditious, and highly sensitive method described here as electron transport chain protocol (ETCP) that demands only a minimal quantity of either live or cryopreserved tissue samples to evaluate Complex I-, Complex II-, and Complex IV-driven mitochondrial respiration in a single Seahorse assay well. Furthermore, we have confirmed the assay sensitivity in discerning brain mitochondrial activity, following TBI. Our study encompasses a comparative analysis of two distinct protocols, a conventional method called oxidative phosphorylation protocol (OxPP) involving freshly isolated mitochondrial samples from the brain and the other method we named ETCP, which utilizes frozen tissue. By scrutinizing fresh *versus* frozen tissue homogenates and isolated mitochondria, we aim to validate and elucidate the effects of tissue preservation on the accuracy and reliability of mitochondrial functional analysis to detect the effects of TBI. This tailored methodology allows for a more refined understanding of mitochondrial function in the context of neurological disorders, particularly after TBI, and opens avenues for enhanced diagnostic and therapeutic approaches in many disease states.

## Methods

### Animal and surgical procedures

All the procedures and protocols were approved by the University of Kentucky Institutional Animal Care and Use Committee and followed the National Institute of Health guide for the care and use of laboratory animals. All data were analyzed and reported as per ARRIVE guidelines. Young adult 8–10-week-old male and female C57BL/6J mice weighing 20–30 g (Jackson Laboratories, Bar Harbor, ME) were housed (5 mice/cage) for 7 days before experimentation to acclimate to the environment and were maintained in a temperature-controlled, 14-h/10-h light/dark cycle room with access to food and water *ad libitum*.

### Controlled cortical impact surgery

All experiments were executed using a total of 28 C57BL/6 mice aged 8–10 weeks (n = 4–6/group; equal numbers of male and female mice) procured from Jackson Laboratories, Bar Harbor, ME (RRID: IMSR_JAX:000,664). The core body temperatures of the mice were maintained at 37°C throughout the surgical procedures and the subsequent recovery period. The surgical protocols have been detailed previously (Singh et al., 2006; Sullivan et al., 2011; Hubbard et al., 2021; Kalimon et al., 2023b). In brief, the mice were anesthetized with 4% isoflurane and affixed within a stereotaxic frame (David Kopf, Tujunga, CA) before the induction of TBI. During the procedural phase, 2.0% isoflurane was administered through a nasal cone. Employing aseptic techniques, the dermal layer was retracted, and a craniotomy of 4-mm diameter was enacted laterally to the sagittal suture, centered between bregma and lambda. The cranial section from the craniotomy was excised without disrupting the subjacent dura. Subsequently, a pneumatically controlled impacting device equipped with a 3-mm-diameter tip was employed to injure the brain, compressing the cortex at 3.5 m/s to a depth of 1.00 mm (resulting in a severe brain injury). For mice undergoing sham surgeries, a craniotomy exposing the cortical surface was performed without impact. Following the injury, ∼4 mm^2^ of the hemostatic dressing, Surgicel (Johnson and Johnson), was applied atop the injury site, and the incision was securely stapled closed. The animals were maintained in clean cages on heating pads at 37°C until complete recovery from anesthesia.

No mortalities were observed from the CCI injury, and no animals were excluded from the study. The mice were randomized for the surgical procedure, and data were analyzed in a blinded manner for the surgical groups. The number of animals per group was predicated based on historical data, wherein n = 4–6 mice per group were employed and a minimum of three technical replicates were utilized for *ex vivo* analysis of mitochondrial bioenergetics (Vekaria et al., 2017).

### Fresh vs. frozen tissue isolation

**FIGURE 1:**
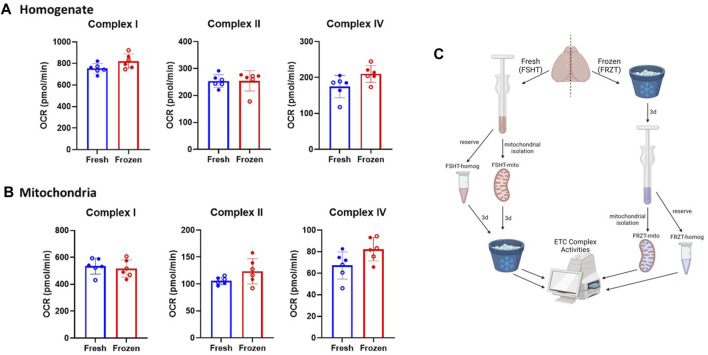
Mitochondrial extraction from frozen stored brain tissue retains the ETC activity: The brains were extracted from naïve males and females (*N* = 6; 3M/3F). using one-half of the freshly isolated brain, **(A)** homogenates, and **(B)** mitochondria were extracted immediately and stored. The other half of the brain was stored at −80⁰C for at least 3 days and was thawed and the homogenates and mitochondrial isolation was carried out as done with the fresh samples. The Seahorse ETCP assay was performed on **(A)** Homogenate fractions and **(B)** mitochondrial fractions of the fresh and frozen tissues. The results indicate there were no significant differences between the complex activities in either homogenates or isolated mitochondria derived from fresh or frozen tissues. **(C)** Schematic representing the workflow of fresh and frozen tissue isolation. OCR values are shown as mean ± SD. Data were analyzed by unpaired t-test.

These experiments were designed to compare ETC complex activities of mitochondria isolated from the fresh tissue immediately following euthanasia and then stored frozen (FSHT) vs. mitochondria that were isolated from tissues that had been previously frozen (FRZT). It is important to note here that although the mitochondria and homogenates are stored frozen after isolation in both cases, it is the tissue that was used fresh or frozen that determines the fresh vs. frozen tag. This terminology will be the same for homogenates as well. For the comparison between fresh and frozen tissues, the whole brains of naïve mice were dissected into two halves on the sagittal plane (Figure 1C). The right hemisphere of each brain was flash-frozen on dry ice and then stored at −80°C, while the remaining left hemisphere was dissected to obtain a combined sample of the cortex and hippocampus (Figure 1C). The isolated mitochondria and reserved homogenates from FSHT tissue (left hemisphere) were then stored at −80°C for 3 days. After 3 days, the FRZT (right) hemisphere was thawed for 2 min in a 37°C water bath, followed by tissue homogenization and mitochondrial isolation.

To compare sham vs. CCI effects on mitochondrial function (Figure 3), 48 h after CCI or sham operation, the ipsilateral side of the injured cortex was dissected using a 4-mm-diameter punch, which was then divided into two equal parts. One half of the tissue punch was flash-frozen on dry ice (FRZT tissue) and stored at −80°C for at least 4 days before carrying out mitochondrial isolation. The remaining fresh tissue (FSHT) was homogenized to get fresh tissue homogenate (FSHT-Homog fraction), following mitochondria isolation protocol to obtain fresh tissue mitochondria (FSHT-Mito fraction). After 4 days, the frozen tissue (FRZT) samples were thawed for 2 min in a 37°C water bath, followed by homogenization to obtain the frozen tissue homogenate (FRZT-Homog fraction), and the mitochondria isolation process was performed in the same way, as done with the fresh tissues to obtain frozen tissue mitochondria (FRZT-Mito fraction). Both the fractions of homogenates and mitochondria isolated from FSHT or FRZT were stored frozen at −80°C until the Seahorse assay was done. To make it clear, although all the homogenate and mitochondrial fractions were frozen at one point, the FSHT-Homog and FSHT-Mito fractions represent the fractions isolated from the freshly isolated tissues. Similarly, FRZT-Homog and FRZT-Mito represent the respective fractions isolated from the stored frozen tissues.

### Mitochondrial isolation from mouse brains

The mitochondrial isolation procedure was carried out using a Ficoll purification method, as reported previously (Kalimon et al., 2023a; Kalimon et al., 2023b). Mice were subjected to brief carbon dioxide euthanasia and decapitated immediately. Brains were removed, and either whole hemispheres (in case of naïve fresh vs. frozen tissue comparison) or the 4-mm ipsilateral cortex punch under the craniotomy site was dissected and homogenized in a Teflon glass Dounce homogenizer in 4 mL or 2 mL mitochondria isolation buffer (MIB; 215 mM mannitol, 75 mM sucrose, 0.1% BSA, 20 mM HEPES, and 1 mM EGTA, adjusted to pH 7.2 with KOH), respectively. All subsequent steps were carried out on ice or at 4°C. The homogenates were transferred to a 2-mL microcentrifuge tube, and from each sample, 100 μL homogenates were collected in a separate 1.5-mL tube containing 1 μL of 100× protease inhibitor cocktail (Halt™ Protease Inhibitor Cocktail (100X); Thermo Fisher # 78430) and reserved for the homogenate bioenergetics analysis. The remaining homogenates were processed for the mitochondrial isolation as follows: the homogenates in the 2 mL tubes were spun at 1,300 ×g for 3 min. The supernatant from the remaining homogenate was then transferred to a fresh 2-mL tube and spun at 13,000 ×g for 10 min. The supernatant was discarded, and the crude mitochondrial pellets were triturated in 400 μL of MIB to attain a final volume of 500 μL. To obtain total mitochondria, it is necessary to release the synaptic mitochondria trapped in synaptosomes after homogenization. The samples were placed into a nitrogen (N_2_) cell disruption chamber at 1,200 psi N_2_ for 10 min at 4°C to burst the synaptosomes and release synaptic mitochondria, followed by loading it over the double layer of Ficoll gradient (2 mL 10% Ficoll on bottom and 2 mL 7.5% Ficoll on top) (Brown et al., 2004; Kalimon et al., 2023a). The Ficoll gradients mounted with crude mitochondria were spun at 100,000 ×g for 30 min, as described previously (Vekaria et al., 2017; Hubbard et al., 2019a). After the spin, the Ficoll solution and debris were carefully removed. The mitochondrial pellets were triturated in 500 μL MIB and were transferred to a 1.5-mL centrifuge tube, and then topped up with the MIB. The mitochondria were pelleted at 13,000 ×g for 10 min at 4°C to obtain a pure mitochondrial pellet. The pellets were resuspended in the MIB (approximately two times the volume of the pellet). The protein concentrations of the homogenate or mitochondrial fractions were determined with a BCA protein assay kit (Pierce, #23227, 2021) by measuring absorbance at 560 nm on the BioTek Synergy HTX multi-mode plate reader (Agilent; RRID: SCR_019749).

### Seahorse oxidative phosphorylation protocol

Bioenergetic analysis of the fresh brain homogenates or purified mitochondria was carried out with OxPP using the Seahorse XFe96 Analyzer (Agilent Technologies; RRID: SCR_019545), as described previously (Hubbard et al., 2021; Kalimon et al., 2023b). In brief, the protein equivalent of 4 μg of the homogenate or 1 μg of the purified mitochondria was loaded on each Seahorse plate well and (n = 6/group with 3–4 technical replicates per sample). Oxygen consumption rates (OCRs) were measured in the presence of various substrates, inhibitors, and uncouplers of the mitochondrial electron transport chain (ETC), as described previously (Hubbard et al., 2018; Hubbard et al., 2021; Kalimon et al., 2023a; Kalimon et al., 2023b). Complex I-mediated State III respiration (respiration coupled to adenosine triphosphate (ATP) synthesis) was measured after the addition of pyruvate and malate (PM; 5 mM pyruvate and 2.5 mM malate), which are tricarboxylic acid (TCA) cycle substrates that feed electrons through Complex I, and adenosine diphosphate (ADP; 2 mM), a necessary substrate to allow the flow of protons through ATP synthase. State IV respiration (proton leak or ATPase-blocked respiration) was measured after the addition of oligomycin (2.5 μM), an inhibitor of ATP synthase. State V(CI) respiration (Complex I-driven maximum/uncoupled respiration) was measured after the addition of FCCP (4 μM), a mitochondrial uncoupler that carries protons back into the matrix, which lowers ΔΨm, and causes the ETC to uncouple from ATP production. Finally, State V (CII) respiration (Complex II-driven maximum/uncoupled respiration) was measured with the addition of rotenone (0.8 μM), a Complex I inhibitor, and succinate (10 mM), a TCA cycle substrate that can feed directly into Complex II of the ETC.

## Seahorse electron transport chain protocol

The homogenates or purified mitochondria either from fresh or frozen brain tissues were utilized for the assessment of mitochondrial electron transport chain complexes in the Seahorse XFe96 Flux Analyzer using ETCP, modified from Jaber et al. (Acin-Perez et al., 2020; Jaber et al., 2020; Kalimon et al., 2023a). Different dilutions of homogenates or purified mitochondria were prepared in mitochondrial respiration buffer with BSA (MRB-w BSA) (125 mM KCl, 0.1% BSA, 20 mM HEPES, 2 mM MgCl_2_, and 2.5 mM KH_2_PO_4_, adjusted to pH 7.2 with KOH). A short hand-out of the assay is described in Table 1. Specifically, protein equivalent, 4 μg of the homogenate, or 0.3 μg of Ficoll purified mitochondria suspended in 75 μL of MRB-w BSA buffer were plated per well and were spun at 3,900 ×g for 10 min at 4°C to pellet mitochondria to the bottom of the well. Meanwhile, the top-up buffer (12.5 mL) was prepared in MRB-w BSA containing 11 ul ionophore alamethicin (stock: 20 mg/mL in ethanol), 440 ul of the coenzyme nicotinamide adenine dinucleotide (NADH) (freshly prepared 100 mM in water), and 55 µL of the electron carrier CytC (4 mM) and was maintained at 37°C. After centrifuging the plate loaded with the homogenate or mitochondria, the wells were very gently topped with 100 μL per well of the top-up solution such that the final concentration of alamethicin was 10 μg/mL, reduced NADH was 30 μM, and cytochrome c was 10 μM. The Seahorse assay was started immediately after topping up without equilibration steps to avoid over-utilization of the NADH substrate. Additional two cycles of mixing steps were added to bring the temperature of the well to 37°C. Remaining substrates and inhibitors of the ETC were prepared in mitochondrial respiration buffer without BSA (MRB -No BSA) and loaded into the injection ports as follows: (A) rotenone (0.8 μM) + succinate (10 mM), (B) antimycin A (1 μM), (C) ascorbate (20 mM) and N,N,N′,N′-tetramethyl-p-phenylenediamine (TMPD; 5 mM), and (D) sodium azide (549.3 mM). In addition, 2–3 OCR readings were taken after each port injection to ensure the full activity was measured. To calculate Complex I activity, the antimycin A reading was subtracted from the first reading. To obtain Complex II activity, the antimycin A reading was subtracted from the rotenone/succinate reading. To obtain Complex IV activity, the sodium azide reading was subtracted from the ascorbate/TMPD reading.

**TABLE 1:** Mitochondrial ETC Complex Protocol (ETCP) for analysis of Frozen Samples Using Seahorse.

1. Prepare the following port solutions per Seahorse plate.			

Injection Port	MRB-No BSA (ul)	Reagent (stock)	Stock vol (μl)
Port A	3220	Rotenone (1 mM) Succinate (1 M)	2.8 280
Port B	3500	Antimycin A (10 mM)	3.5
Port C	3325	Ascorbate (0.8 M) TMPD (0.2 M)	88 88
Port D	3033	Sodium Azide (4 M)	480
2. Load the Seahorse cartridge with 25 μl per injection port with the respective port solutions			
3. Start the Seahorse plate calibration with a 30-sec mix, 2 min read, and 2 cycles per step protocol			
4. Load a Seahorse culture plate with 75 μl (0.3 ug brain mitochondria or 4 ug brain homogenate) mito samples per well resuspended in MRB wBSA.			
5. Balance and spin the Seahorse plate at 3900xg for 10 minutes at 4°C.			
6. Prepare 12.5 ml of the top-up solution per plate (keep it incubating at 37°C before loading) and top up 100 μl per well gently			

Reagent (Final conc.)	Stock	Stock vol per ml	Vol for 12.5 ml (For 1 plate)
Alamethicin (10 ug/ml)	20 mg/ml	0.9 μl	11 μl
*NADH (30 uM)	100 mM	35 μ	440 μl
Cyt C (10 uM)	4 mM	4.4 μl	55 μl
MRB w BSA (37°C)		1 ml	12 ml
*Prepare NADH fresh as per need (71 mg/ml in H_2_O)			
7. Load the Culture plate immediately after top-up and start the assay			

### Western Blot

The FSHT-Mito, FSHT-Homog, FRZT-Mito, and FRZT-Homog samples were diluted in the XT protein loading dye (Bio-Rad Cat. #1610791) and were separated on a 4%–12% SDS-PAGE (Bio-Rad Cat. #3450125) and transferred to a nitrocellulose membrane. The proteins were probed for all five ETC complexes using the total OXPHOS antibody cocktail (Abcam Cat. #ab110413), and the bands were normalized to the total protein using the No-Stain™ Protein Labeling Reagent kit, according to the manufacturer’s instructions (Thermo Fisher Cat. #A44449).

## Results

### Single-well Seahorse assay (ETCP) for comprehensive ETC function analysis for frozen mitochondria

**FIGURE 2:**
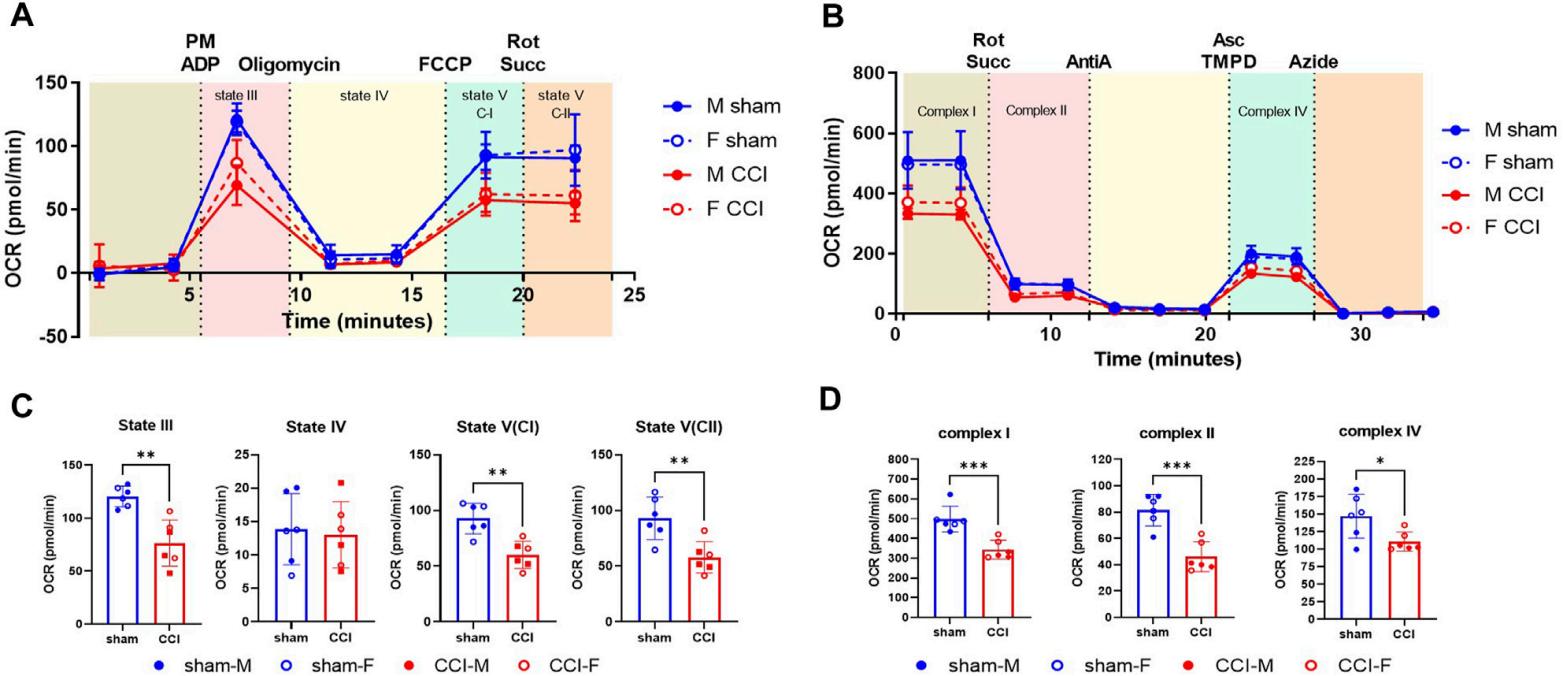
OxPP and ETCP protocols show the mitochondrial function deficit after CCI: The male and female mice were subjected to sham or CCI injury (*N* = 6; 3M/3F). At 48h post-injury, the mitochondria were isolated from the injured cortex (4mm punch) using ficoll purification. The mitochondrial bioenergetics were assessed for **(A)** intact mitochondria using oxphos protocol (OxPP) and **(B)** Alamethicin permeabilized mitochondria using ETC protocol (ETCP). Results indicate that comparing the two protocols, there were similar levels of injury effects between sham and CCI groups **(C, D)** while comparing the states of respirations in OxPP to that of complex activities in ETCP, indicating the validity of the ETC protocol for the use of frozen samples. OCR values are shown as mean ± SD. Data were analyzed by unpaired t-test. *p < 0.05; **p < 0.01; ***p < 0.001.

Here, we have modified a recently developed method for the mitochondrial function analysis and adapted the method to make the best use of the injection ports in Seahorse mitochondrial functional analysis (Acin-Perez et al., 2020; Jaber et al., 2020). To assess all three complexes in the same Seahorse well and to carry out the assay with a limited sample in ETCP, reagents were utilized to measure Complex I, Complex II, and Complex IV activities in a single seahorse culture plate well (see Table 1), thereby making the ETCP more efficient and sensitive. As shown in Figure 2B, the Complex I reagents were added just before loading the Seahorse assay plate into the machine to measure Complex I activity. The first injection, port A, injected rotenone to inhibit Complex I and succinate to feed Complex II via succinate dehydrogenase to measure Complex II-driven respiration. After measuring Complex II activity, antimycin A was injected through port B to inhibit Complex III activity. The antimycin A rate was used to determine the baseline for complexes I and II. The injection port C injected ascorbate and TMPD to feed the electrons through Complex IV, measuring the Complex IV respiration. Complex IV activity was inhibited using the azide injection via port D to get a baseline for Complex IV. This sequential approach allows for the measurement of ETC complexes I, II, and IV activities in a single well to determine the mitochondrial function. To test the efficacy of this protocol using frozen samples, we compared the fresh and frozen tissue samples (Figure 1). The brains were extracted from naïve mice, and one hemisphere was flash-frozen and then stored at −80°C. The other half of the brain was used to extract the homogenate and mitochondria, which were then stored at −80°C (Figure 1C). After 3 days, the mitochondria and the homogenates were isolated from the frozen half-tissue samples. All the homogenates and mitochondrial fractions (Figures 1A, B, respectively) were run simultaneously on the same Seahorse plate, and the rates were compared. It was observed that there were no significant differences in ETC complex activities between fresh and frozen samples, indicating that the mitochondrial ETC flux activities are maintained in the frozen samples from naïve mice.

### Comparative ETCP and OxPP protocols to establish mitochondrial dysfunction after CCI

As a determining factor for neurodegeneration, mitochondrial respiration assessment is one of the most reliable parameters utilized in preclinical models including Alzheimer’s disease, Parkinson’s disease, TBI, and SCI (Palacino et al., 2004; Singh et al., 2006; Patel et al., 2009; Yao et al., 2009; Prasuhn et al., 2020; Rabchevsky et al., 2020; Mi et al., 2023). Here, we have used the severe CCI injury model to validate the use of the ETC assay developed in our laboratory as an indicator of mitochondrial function analysis in frozen samples. To compare the fresh vs. frozen effects on the mitochondrial function determination, male and female mice were subjected to sham or CCI injury, as mentioned in the Methods. The injured cortical region was excised using a 4-mm-diameter punch 48 h after injury and then subjected to mitochondrial isolation using the Ficoll method (Figure 2C). To compare the two protocols, in this particular experiment, the mitochondria were isolated from the fresh brain tissue and were used immediately after the isolation. The freshly isolated mitochondria were assessed using a previously established OXPHOS protocol (OxPP) (Figure 2A) and the newly developed ETC complex activity protocol (ETCP) (Figure 2B). It was observed that in the OxPP assay, Complex I-mediated coupled respiration (State III) and uncoupled respiration (State V(CI)) and Complex II-mediated uncoupled respiration State V (CII) were significantly decreased in the CCI group, as compared to the sham group (Figure 2C). The ETCP protocol was run in parallel with the OxPP protocol and demonstrated similar effects on mitochondrial respiratory function. It was observed that the Complex I, Complex II, and Complex IV activities were decreased significantly in the CCI group, as compared to the sham group (Figure 2D). This comparative analysis suggests that the OxPP and ETCP protocols are suitable for mitochondrial function determination after TBI and can help determine the ETC function in other neurodegeneration models as well.

### Validation of ETCP protocol in fresh vs. frozen brain tissue samples after CCI

**FIGURE 3:**
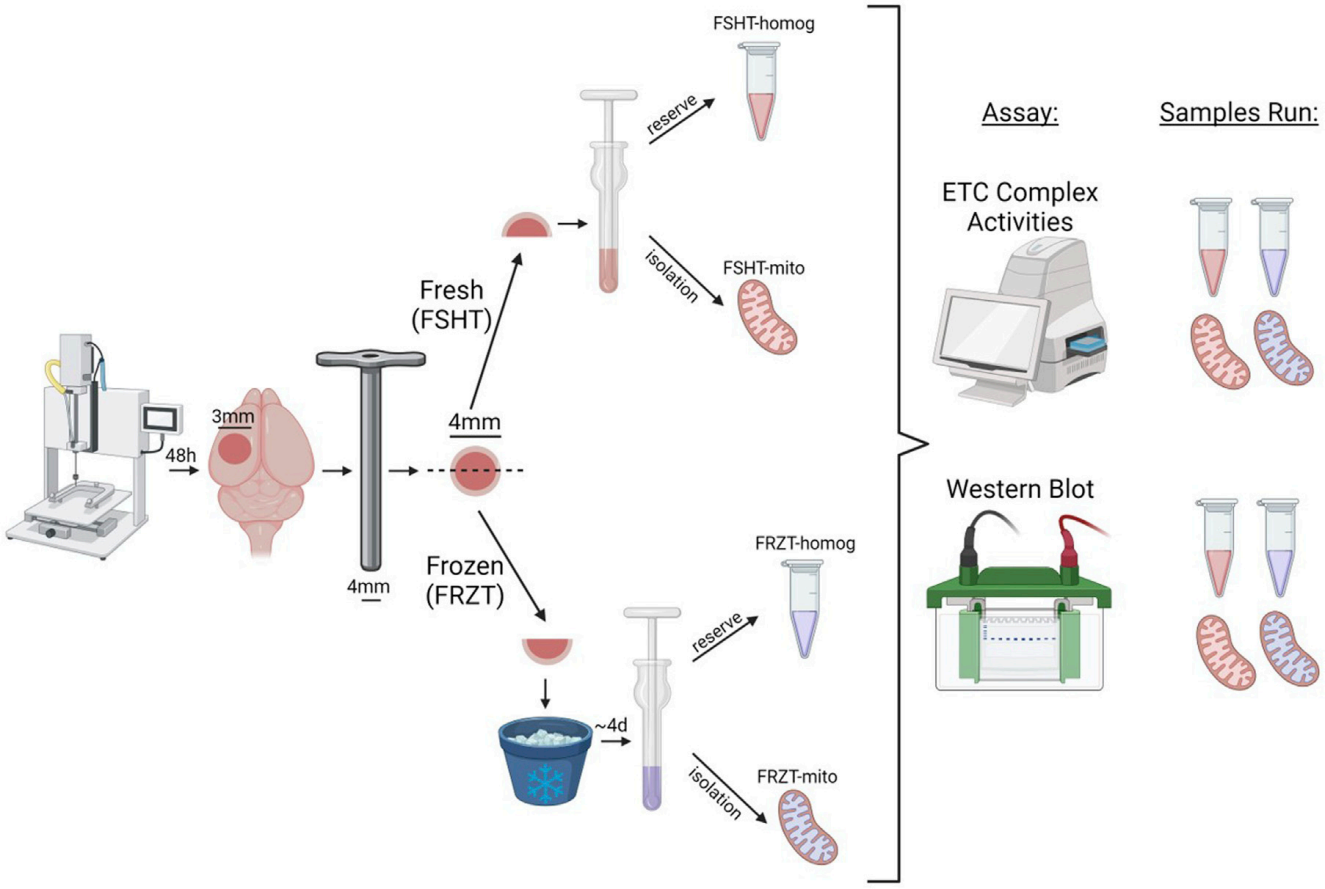
Schematic workflow of CCI injury, Tissue collection, sample preparation, and storage. The male and female mice were subjected to sham or CCI injury. The ipsilateral injured cortex punch (4 mm) was split into two equal parts. One fraction was snap frozen on dry ice and stored at −80⁰C and the remaining one was used for fresh homogenate and mitochondria isolation and was labeled as FSHT-Homog and FSHT-Mito respectively. After 4 days the frozen cortices were thawed at 37°C for 2 minutes and were used for the collection of frozen homogenates and mitochondria and labelled as FRZT-Homog and FRZT-Mito respectively. The fractions were first analyzed for Electron Transport Chain function analysis using ETCP protocol and later for the Western blot.

**FIGURE 4:**
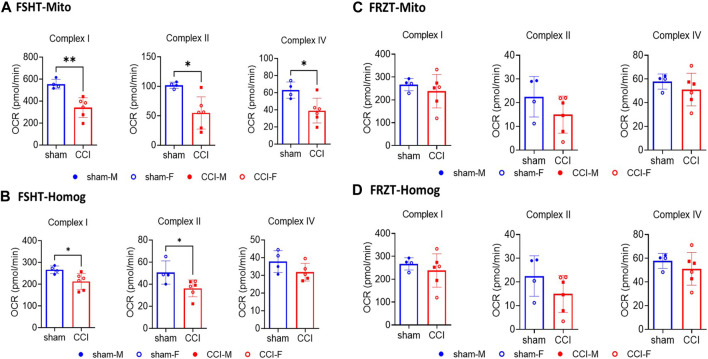
Seahorse ETC complex activities in mitochondria and homogenates isolated from fresh or frozen cortex tissues: The male and female mice were subjected to sham or CCI injury (*N *= 4-6; Equal males and females). As illustrated in Figure 3, the ipsilateral injured cortex punch (4mm) was split into two equal parts. One fraction was snap frozen and stored at -80⁰C and the remaining one was used for fresh mitochondria (FSHT-Mito) and homogenate (FSHT-Homog) preparation **(A, B)**. Using the ETCP protocol, the complex I, II, and IV activities in mitochondria and complex I and II activities in homogenates showed a significant reduction in CCI as compared to sham animals. Whereas the frozen cortices’ mitochondria (FRZT-Mito) and homogenates (FRZT-Mito) prepared **(C, D)** after 4 days didn’t show any significant differences between the sham and CCI group of animals. OCR values are shown as *N *= 4-6 ± SD. Data were analyzed by unpaired t-test. * *p *< 0.05, ** *p *< 0.01.

To investigate the validity of the ETCP assay for the assessment of mitochondrial function in frozen samples, we carried out mitochondrial functional analysis of the homogenates and isolated mitochondria from freshly isolated tissues and the frozen tissues. To carry out this comparative analysis, the mice were subjected to a severe (1.00 mm depth) CCI injury. At 48 h post-injury, the injured core and surrounding penumbra of the ipsilateral cortex were excised out using a 4-mm-diameter punch, and each tissue punch was split into two equal halves (see Figure 3 for the workflow). One half of the punch was flash-frozen on dry ice and stored at −80°C. The other half was homogenized immediately, as done in the previous experiment, into 2 mL MIB. The 200 μL homogenate was aliquoted, and the remaining was processed for mitochondrial isolation. All the samples (i.e., frozen tissue, homogenate, and isolated mitochondria) were stored at −80°C until the assay was done. After 4 days, the frozen cortex tissue was thawed at 37°C for 2 minutes and was then homogenized in 2 mL MIB. The homogenates were preserved, and mitochondria were isolated in the same way, as from the fresh tissues described above, and were stored frozen until the assays were done (see Figure 3 for the workflow). All four fractions categorized in methods, i.e., FSHT-Homog, FSHT-Mito, FRZT-Homog, and FRZT-Mito, were assayed for the ETC activities, as mentioned previously. The results indicate that FSHT-Mito (Figure 4A) and FSHT-Homog (Figure 4B), which were stored frozen after the isolation, still demonstrated the injury effect for complex I and II activities. Only the mitochondria showed significantly decreased Complex IV activity, while the homogenates did not show the injury effect for Complex IV activity (Figure 4A, B). Overall, the injury effect was more prominent in freshly isolated mitochondria, showing approximately 50% reduction in activities in CCI groups compared to sham, whereas the homogenates showed comparatively less injury effects. Analyzing the frozen tissue-derived mitochondria (Figure 4C) and homogenates (Figure 4D), there were no significant differences observed between sham and CCI groups in FRZT-Mito or FRZT-Homog samples. Although the isolated mitochondria showed some downward trend, overall, both the homogenates and isolated mitochondria from frozen tissues showed no differences between sham and CCI groups.

### Effect of CCI injury on ETC complex protein expression

**FIGURE 5:**
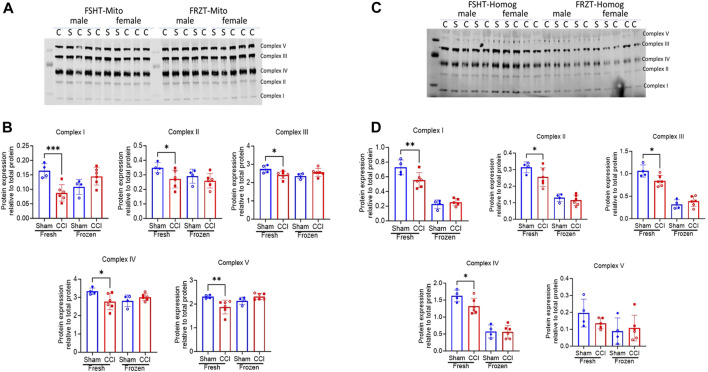
Western blot mitochondrial complex protein expression in mitochondria and homogenates isolated from fresh or frozen cortex tissues: As illustrated in Figure 3, the male and female mice were subjected to sham or CCI injury (*N *= 4-6; Equal male and females). The ipsilateral injured cortex punch (4mm) was split into two equal parts. One fraction was snap frozen and stored at −80⁰C and the remaining one was used for fresh mitochondria and homogenate preparation. The extracts were lysed in RIPA buffer and were analyzed for protein expression using Western Blot analysis using an Oxphos antibody cocktail (Abcam #ab110413). All 5 complexes in mitochondria (FSHT-Mito) from fresh tissues showed a significant reduction in CCI as compared to sham animals (A Left panel). Whereas the mitochondria (FRZT-Mito) prepared from frozen cortices (A right panel) after 4 days didn’t show any significant differences between the sham and CCI group of animals. Similarly, homogenates (FSHT-Homog) (C Left panel) prepared from fresh tissues showed a significant decrease in complexes I, II, III, and IV expression but there were no changes in the (FRZT-Homog) homogenates prepared from the frozen tissues (C right panel). (B and D show the quantitative analysis of figures A and C respectively. Protein expression data are presented as *N *= 4-6 ± SD. Data were analyzed by one-way ANOVA with Fisher’s LSD test. * *p*,0.05, ** *p *< 0.01, *** *p *< 0.001.

To further investigate the loss of ETC activities in the FRZT-derived homogenates and mitochondria against FSHT-derived homogenates and mitochondria, we processed the remaining homogenates and isolated mitochondria for Western blot analysis of proteins involved in oxidative phosphorylation. The Western analysis showed a similar pattern as the ETCP complex activity assay. There was a 50% reduction in the Complex I protein expression in FSHT-Mito from the CCI-injured mice compared to the sham group (Figure 5A, B). Other complex protein expressions, i.e., complexes II, III, IV, and V, were also decreased in CCI groups, as compared to the sham group (Figure 5A, B). Similarly, in homogenate fractions, there was a decrease in the complex I, II, III, and IV protein expressions in the FSHT-Homog in CCI groups, as compared to the sham groups (Figure 5C, D). In comparison, FRZT-Homog and FRZT-Mito isolated from the frozen tissues did not show any significant changes between the sham and CCI groups (Figure 5A–D) for any of the five complexes, thereby confirming the results in ETCP complex activity determinations. These results indicate that the mitochondrial function is well-preserved in the fresh homogenates and mitochondria that were stored frozen, as compared to the whole tissue storage at −80°C.

## Discussion

Mitochondria play a central role in eukaryotic cell metabolism. Being a major energy producer, they are very sensitive to the local environment, and depending on the energy demand and supply requirements of a cell, they undergo considerable modifications in morphology and function (Brown et al., 2006; Datta and Jaiswal, 2021; Joubert and Puff, 2021). Considering these continuous changes in mitochondrial function, they serve as one of the most important markers of cellular physiology.

Mitochondrial functional analysis has become one of the gold standards for the evaluation of pathobiology that occurs in preclinical models of TBI and other neuronal degenerative diseases. The conventional approach to study mitochondrial bioenergetic flux is to use a Clark-type electrode which measures the oxygen (O_2_) consumption rate, following sequential addition of various substrates/inhibitors/uncouplers in a continuously stirred, sealed chamber (Pandya et al., 2009). Recent advancements in the fluorescent-based O_2_ determination methods using Seahorse technology have revolutionized the field with a high-throughput measurement of mitochondrial O_2_ consumption rates in a variety of cells and tissues (Sauerbeck et al., 2011; Schuh et al., 2011). Historically, respirometric flux experiments have been restricted to utilization in fresh biological samples. Recently, efforts have been made to carry out partial mitochondrial activity assessments in the frozen samples. Jaber *et al.* showed that mitochondrial function in a cell culture system can be analyzed beyond the widely used Mito Stress Test protocol using NADH as a direct substrate for Complex I (Jaber et al., 2020). This method can be useful in testing the drugs targeted against ETC function. A study carried out by Parez *et al.* showed that the frozen tissues retain mitochondrial-specific membrane ETC complex activities (Acin-Perez et al., 2020). Here, we have improved upon these methods and adapted them in analyzing mitochondrial-specific complex I, II, and IV-driven respiration in a single well of the 96-well plate to use the assay in a high-throughput manner, requiring a much lower amount of sample compared to other methods. Furthermore, we validated the use of the assay after the CCI model of TBI and evaluated the advantages and limitations to using frozen tissues, homogenates, and isolated mitochondria, which are discussed below. These methods can further be extrapolated to determine the mitochondrial function in other disease models such as Alzheimer’s disease, Parkinson’s disease, and spinal cord injury.

As mentioned, we have evaluated this method to study the mitochondrial dysfunction in the CCI injury model of TBI. Previous studies conducted by our group have demonstrated that as a consequence of secondary injury after CCI, mouse cortical mitochondria experience a significant impairment, and therapies that target mitochondrial dysfunction can have a neuroprotective effect (Singh et al., 2006; Vekaria et al., 2020; Kalimon et al., 2023b). In the present study first using freshly isolated mitochondria, we demonstrated that State III, State V (CI), and State V (CII) decreased significantly 48 h after injury compared to the sham group. To validate the similar mitochondrial dysfunction using the, ETC protocol, first, we compared the two protocols, named OxPP and ETCP on freshly isolated mitochondrial samples because we cannot perform OxPP in frozen samples. Specifically, the two protocols are designed based on the sample characteristics of the mitochondria to be analyzed. OxPP strictly requires intact mitochondria with complete membrane integrity and gives a comprehensive mitochondrial flux analysis by generating the membrane potential and having all the matrix enzymes to couple the TCA cycle, ETC, and ATP synthase reactions (Sauerbeck et al., 2011). In comparison, the frozen samples have compromised membrane integrity due to freeze–thaw-associated membrane disruption, leading to a lack of membrane potential to generate ATP, and due to the same reason, diffusion of matrix enzymes, leading to inefficient TCA cycle-mediated substrate-driven respiration (Nukala et al., 2006; Acin-Perez et al., 2020). To overcome these limitations, the TCA cycle can be bypassed by directly feeding electrons into the ETC using NADH or succinate as substrates (Jaber et al., 2020). Usually, with intact mitochondria, NADH is impermeable to the inner mitochondrial membrane and cannot be used as a substrate for Complex I (Stein and Imai, 2012; Kharechkina et al., 2021). So the inner mitochondrial membrane is completely permeabilized using a pore-forming peptide, alamethicin, which makes a channel in the membrane, allowing free transfer of substrates for ETC (Mathew et al., 1981). Another limitation of using frozen samples is the loss of the outer mitochondrial membrane, which leads to diffusion of cytochrome C which is the only free component of the ETC residing in the intermembrane space and limits the flow of electrons to oxygen (Martinou et al., 2000; Sullivan et al., 2002; Nukala et al., 2006). To overcome this, cytochrome C is added in excess to allow ETC flux through Complex IV. All these modifications led to the successful analysis of mitochondrial ETC complex activity flux in frozen samples. Our comparative analysis of the two protocols (i.e., OxPP and ETCP) successfully demonstrated a mitochondrial bioenergetic deficit 48 h after CCI. We show from the ETCP that the activities of complexes I, II, and IV were compromised in the CCI-injured mitochondria, as compared to that from the sham-operated animals, which are comparable to State V (CI) and State V (CII) respirations in OxPP.

Mitochondrial isolation involves homogenization and centrifugation steps which may have variable impacts on the processing of fresh or frozen tissues. To determine the impact of freezing and processing of the frozen tissue on the mitochondrial ETCP bioenergetics, we compared the homogenates and mitochondria isolated from fresh and frozen tissues and analyzed them using the ETCP. It is important to note that although all the mitochondria and homogenate fractions were frozen after the isolation, the major difference is that in the first case (FSHT), the mitochondria and homogenates were prepared from fresh tissues and then stored. However, in the second case (FRZT), they were prepared from previously frozen tissues and stored. We found that both, FSHT-Homog and FSHT-Mito isolated immediately from the fresh tissues, still demonstrated the injury effect. FSHT-Mito from the CCI group showed deficits in the activities of complexes I, II, and IV, as compared to the sham group (Figure 4A). The FSHT-Homog samples also showed significant decreases but had a comparatively lower effect than pure mitochondria in Complex I and Complex II activities (Figure 4B). There were no impairments in Complex IV activities in the CCI group, as compared to their respective sham (Figure 4B). In addition, we showed that isolated mitochondria showed a more prominent ETC function, as compared to the homogenate. So, mitochondrial isolation would be ideal when looking at the subtle differences in mitochondrial function across disease or injury states.

In comparison, with homogenates and mitochondria prepared from previously frozen tissues (FRZT), we found that there were no significant differences between sham and CCI groups in either FRZT-Mito or FRZT-Homog samples with any of the three complex activities measured. This suggests that freezing the whole brain tissue and then isolating mitochondria introduce additional damage to the respiratory complexes I and II when compared to mitochondria isolated from the fresh tissue samples. This is surprising since we demonstrated that the mitochondria isolated from the frozen tissues retained the ETC activities (Figures 1A, B). There could be many possible reasons for this discrepancy. The most important difference could be the way the fresh and frozen samples are processed in the homogenization process. Usually during homogenization, mitochondria remain intact due to their small size, whereas in frozen samples, they get lysed easily. Special care should be taken when homogenizing frozen tissues, ensuring tissues are completely thawed so that ice crystals do not further lyse the sample. Another difference is the change in the buoyancy due to the inner mitochondrial membrane disruption. This density change could vary the sedimentation rate upon centrifugation and can result in loss of mitochondria during isolation, which may favor a loss of damaged mitochondria.

To investigate whether the activities are correlated with protein expression, we measured the protein expression of the ETC complexes using Western blot (Figure 4). We observed a very similar pattern as observed with the ETCP assay measurements. The mitochondrial protein expression of all five ETC complexes, (i.e., complexes I–IV of ETC and Complex V or ATPase) was decreased in the CCI group, as compared to sham, in FSHT-Homog and FSHT-Mito samples (Figure 4A, B). The mitochondria and homogenates prepared from the frozen samples, i.e., FRZT-Homog and FRZT-Mito samples, however, showed no difference between sham and CCI groups.

Overall, here, we propose a single-well assay format and improved ETCP method to analyze the mitochondrial ETC function in frozen homogenates or mitochondrial samples that allow for the simultaneous measurement of mitochondrial flux driven with the substrates of complexes I, II, and IV in a single assay. The assay is very sensitive, requiring a very small amount of the sample to run a high-throughput analysis. This assay may help carry out the mitochondrial functional analysis in clinical tissues in different pathological conditions. The results also suggest that it is better to process the freshly isolated tissues and prepare homogenates and isolated mitochondria fractions for storage in contrast to storage of the intact tissues when estimating the mitochondrial function.

Limitations: This protocol is suitable for measuring the mitochondrial ETC complex activities in a high-throughput manner, and it is not proposed to replace the comprehensive functional mitochondrial flux, as done with the fresh mitochondria. Chiefly, one cannot measure the mitochondrial membrane potential and leak respiration as the membrane integrity is compromised in frozen samples. The method cannot give information about the TCA cycle flux as this protocol uses NADH and succinate directly to feed the ETC and bypass the TCA cycle. Still, the method can be very helpful in providing complementary information and mechanistic waypoints of mitochondrial dysfunction after TBI and can be extrapolated in other pathophysiologic conditions.

## Data Availability

The original contributions presented in the study are included in the article/Supplementary Material; further inquiries can be directed to the corresponding authors.

## Abbreviations

TBI, traumatic brain injury; CCI, controlled cortical impact; MIB, mitochondrial isolation buffer; MRB-w BSA, mitochondrial respiration buffer with BSA; MRB-NoBSA, mitochondrial respiration buffer without BSA; ETC, electron transport chain; OXPHOS, oxidative phosphorylation; FSHT-Homog, fresh tissue homogenate; FSHT-Mito, fresh tissue mitochondria; FRZT-Homog, frozen tissue homogenate; FRZT-Mito, frozen tissue mitochondria; ETCP, electron transport chain protocol; OxPP, oxidative phosphorylation protocol; TCA cycle, tricarboxylic acid cycle.
